# 

**Published:** 1896-06

**Authors:** 


### TABLE OF CONTENTS.

ORIGINAL COMMUNICATIONS. PAGE

Electrical Osmosis in the Treatment of Acute Dentinal Sensibility. By Louis Jack, D.D.S. . . . . 341

On "Light." By Myles Standish, M.D., Boston, Mass. . . . . 345

Some of the Physical Properties of Metals. By Professor A. A. Breneman . . . . . 355

The Advantages of Cataphoresis in Dental Operations. By Peter Brown, L.D.S., Montreal . . . . . 365

An Interesting Case. By Dr. S. L. Goldsmith, New York . . . . . 370

Lighting the Mouth. By William H. Rollins, Boston, Mass. . . . . . 371

Third Set of Teeth . . . . . 371

TRANSLATIONS.

Solila. By Theodore Frick, D.D.S., Switzerland . . . . . 372

REPORTS OF SOCIETY MEETINGS.

American Academy of Dental Science . . . . . 376

The New York Institute of Stomatology . . . . . 385

EDITORIAL.

After-Thoughts . . . . . 390

Electrical Osmosis . . . . . 393

Solila Gold . . . . . 394

A Journalistic War . . . . . 395

BIBLIOGRAPHY . . . . . 395

OBITUARY.

Dr. Phineas G. C. Hunt . . . . . 399

Paul Dubois . . . . . 400

Dr. Leonard R. Koecker . . . . . 401

Resolutions of Respect—Dr. W. H. Dwinelle . . . . . 401

CURRENT NEWS.

Pennsylvania State Dental Society . . . . . 402

Pennsylvania State Dental Examining Board . . . . . 402

Chicago Dental Society . . . . . 402

North Carolina State Dental Society . . . . . 403

American Dental Society of Europe . . . . . 403

The Fifty-first Anniversary of Horace Wells's Discovery . . . . . 403

Interstate Dental Meeting . . . . . 404

